# Transcriptome analysis reveals the role of the root hairs as environmental sensors to maintain plant functions under water-deficiency conditions

**DOI:** 10.1093/jxb/erv498

**Published:** 2015-11-19

**Authors:** Miroslaw Kwasniewski, Agata Daszkowska-Golec, Agnieszka Janiak, Karolina Chwialkowska, Urszula Nowakowska, Gaurav Sablok, Iwona Szarejko

**Affiliations:** ^1^Department of Genetics, University of Silesia in Katowice, 40-032 Katowice, Poland; ^2^Plant Functional Biology and Climate Change Cluster, University of Technology, Sydney, Ultimo, NSW 2007, Australia

**Keywords:** Barley, drought, environmental sensor, gene expression, root hair, water stress.

## Abstract

An important part of the root system is the root hairs, which play a role in mineral and water uptake. Here, we present an analysis of the transcriptomic response to water deficiency of the wild-type (WT) barley cultivar ‘Karat’ and its root-hairless mutant *rhl1.a*. A comparison of the transcriptional changes induced by water stress resulted in the identification of genes whose expression was specifically affected in each genotype. At the onset of water stress, more genes were modulated by water shortage in the roots of the WT plants than in the roots of *rhl1.a*. The roots of the WT plants, but not of *rhl1.a*, specifically responded with the induction of genes that are related to the abscisic acid biosynthesis, stomatal closure, and cell wall biogenesis, thus indicating the specific activation of processes that are related to water-stress signalling and protection. On the other hand, the processes involved in the further response to abiotic stimuli, including hydrogen peroxide, heat, and high light intensity, were specifically up-regulated in the leaves of *rhl1.a*. An extended period of severe stress caused more drastic transcriptome changes in the roots and leaves of the *rhl1.a* mutant than in those of the WT. These results are in agreement with the much stronger damage to photosystem II in the *rhl1.a* mutant than in its parent cultivar after 10 d of water stress. Taking into account the putative stress sensing and signalling features of the root hair transcriptome, we discuss the role of root hairs as sensors of environmental conditions.

## Highlight

The root system is the first plant organ that senses a water deficit. Here we have shown evidence that root hairs play a role as sensors of environmental conditions during water stress.

## Introduction

The root system is the first plant organ that senses a water deficit, and it is well known that the root system architecture changes in response to drought (Sharma and Ghildyal 1977; Huck *et al.*, 1983). A water deficit often results in an increased root/shoot ratio (Sharp *et al.*, 2004) and influences the root system plasticity (Ehdaie *et al.*, 2012). Root hairs—the tubular-shaped outgrowths of root epidermis cells—are an important part of the root system. The mechanisms of their development are relatively well described in Arabidopsis (reviewed by Carol and Dolan, 2006; Kwasniewski *et al.*, 2013a), and some molecular data regarding root hair formation in monocot species are also available (Wen *et al.*, 2005; Kwasniewski and Szarejko, 2006; Hochholdinger *et al.*, 2008; Yuo *et al.*, 2009, 2011; Kwasniewski *et al.*, 2010; Janiak *et al.*, 2012; Huang *et al*., 2013a, b; Marzec *et al.*, 2015). It is generally accepted that root hairs play an important role in water and mineral uptake and may help in the anchorage of a plant in the soil. They are also the site of a plant’s interactions with soil micro-organisms. The literature data on the role of root hairs in mineral uptake is relatively well documented, especially in the case of phosphorous. On the other hand, their role in water uptake is intuitively understood, but direct experimental proof of such a function is limited. There are no experimental data available that specifically link the presence of root hairs with the general response of a plant to water deficits, as measured at molecular levels. As has been shown by mathematical models, root hairs significantly increase the root surface area, which may play a role in water and nutrient uptake, especially those with a low mobility in soil, such as phosphorous and potassium (Jungk, 2001; Leitner *et al.*, 2010). Nutrient uptake results in a rapid decrease in its concentration around the root and thus leads to soil depletion (Jungk and Claassen, 1997). At the same time, the growth of the root and the fast rate of root hair replacement, which was calculated in barley (*Hordeum vulgare* L.) as 40–50h (McElgunn and Harrison, 1969), causes the continuous movement of the root hair zone through the soil into areas where nutrients are still available (Jungk, 2001). The observation of a correlation between the length and density of root hairs and the availability of a particular nutrient in the soil provides another piece of evidence of their role in ion sensing and transport. Both root hair length and density increased during the exposure of barley plants to phosphorous starvation (Gahoonia and Nielsen, 1998) or iron deficiency (Schmidt and Schikora, 2001; Muller and Schmidt, 2004).

Before any mechanism of systemic adaptation can be activated in response to drought stress, the plant must first perceive the signal of a water deficit in the soil. It is widely accepted that the first steps of the sensing and signalling of water deficit conditions involve the mechanisms of an osmotic stress response (Urao *et al.*, 1999), reactive oxygen species (ROS)-mediated signalling (Miller *et al.*, 2010; Kar, 2011), and the activation of hormone-dependent transcriptomic cascades, along with the predominant role of abscisic acid (ABA) (Schachtman and Goodger, 2008). Interestingly, root hairs have been shown to respond to drought or osmotic stress by disrupting tip elongation and becoming swollen (Dauphin *et al.*, 2001; Volgger *et al.*, 2010). ABA-mediated drought signalling triggers a signal transduction cascade that involves phospholipids and calcium-dependent kinases. These groups of proteins are also involved in root hair development (Jones *et al.*, 2002; Oyama *et al.*, 2002; Xu *et al.*, 2005); however, no evidence of any interplay between sensing/signalling of water stress and root hair formation has been discussed to date.

In our previous studies, a completely root-hairless mutant, *rhl1.a*, and its wild-type (WT) parent cultivar ‘Karat’ were used extensively for identification of the genes that are involved in root hair development in barley (Kwasniewski and Szarejko, 2006; Kwasniewski *et al.*, 2010; Janiak *et al.*, 2012; Kwasniewski *et al.*, 2013b; Chmielewska *et al.*, 2014). Here, we used our model system of the root-hairless mutant *rhl1.a* and its WT parent cultivar ‘Karat’ to elucidate for the first time the potential role of root hairs as the environmental biosensors of water availability, thereby aiding in the maintenance of proper plant functions under water-deficiency conditions. Global transcriptome analysis, which was carried out in the roots and leaves of a root-hairless mutant and its WT parent during a time-course drought experiment, complemented by a physiological analysis of photosynthesis activity, provide original insights into the mechanisms that are involved in the root hair-dependent response to water stress in barley.

## Material and methods

### Plant material

A root-hairless mutant, *rhl1.a*, obtained in the Department of Genetics, University of Silesia, Poland, through *N*-methyl-*N*-nitroso urea treatment of the spring barley variety ‘Karat’ that produces normal root hairs, was used in this study. As was previously reported, the root-hairless phenotype of the *rhl1.a* mutant is controlled by a single recessive gene (Szarejko *et al.*, 2005) that is allelic to the root-hairless mutant *brb* (*bald root barley*; Gahoonia *et al.*, 2001). In the *rhl1.a* and *brb* mutants, the epidermal cells are homogeneous with respect to both their length and cytoplasm density, thus indicating that the root-hairless phenotype is caused by the lack of asymmetric cell expansion, which is observed in the WT plants (Marzec *et al.*, 2013). The mutant was backcrossed twice to its parent variety to clean the genetic background of other mutations. BC_2_F_6_ seedlings from the cross of *rhl1.a*×‘Karat’, which were classified according to their root hair phenotype (mutant or WT), were used for all transcriptome and physiological analyses.

### Preliminary analysis of root hair formation transcriptome: growth conditions and material sampling

Studies on the identification of differentially expressed genes (DEGs) in the roots of the *rhl1.a* mutant versus ‘Karat’ were carried out as described previously (Kwasniewski *et al.*, 2010) using 1cm long root tip fragments that contained tissues from different root zones, including the root cap, meristem, root elongation, and root hair differentiation zone; however, the major part of the sample comprised tissues from the mature root hair zone. Seeds were surface sterilized with a 20% (v/v) bleach solution and germinated in sterile aeroponic conditions on a wet cotton wool plug, trapped between two glass tubes, which were linked with Parafilm M (Pechiney Plastic Packaging). Six-day-old seedlings were used for RNA isolation.

### Water-deficiency stress experiments

#### Water-stress treatment

**Fig. 1.**
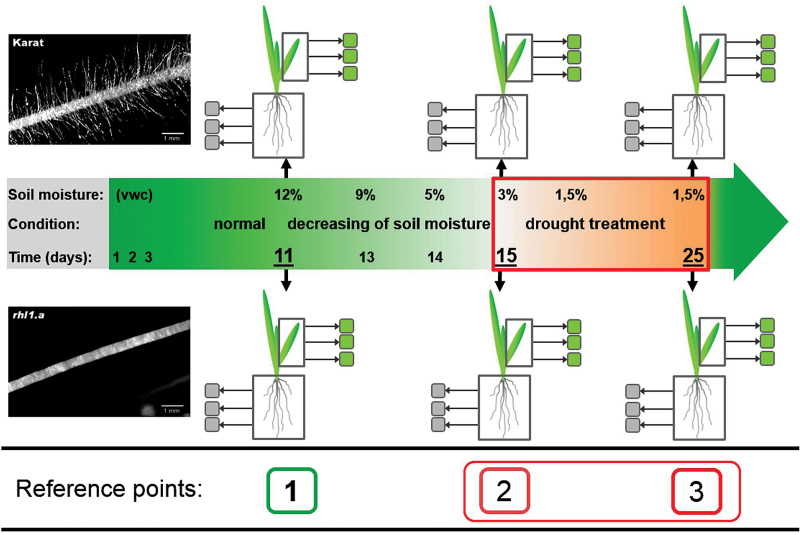
Water-stress experiment carried out on the WT cultivar ‘Karat’ and its root-hairless mutant *rhl1.a* (inset photos). The reference points that are discussed in the text are indicated as: (1) 11 DAS, with normal conditions and soil moisture of 12%; (2) 15 DAS, with soil moisture decreased gradually to 3%, resulting in the onset of drought stress; and (3) 25 DAS, with soil moisture of 1.5%, after 10 d of severe drought.

Water stress was applied in controlled conditions during the seedling stage. First, seeds were sowed in Petri dishes, which were filled with water-soaked vermiculite and kept at 4 °C in darkness for 2 d, and were then moved to 20 °C in a greenhouse for another 2 d. In the meantime, pots (400×140×175mm in size) were filled with soil with known physicochemical properties, which was composed of sandy loam and sand (7:2 ratio) and supplemented with a nutrient medium [428.5mM NH_4_NO_3_, 299.8mM KH_2_PO_4_, 57.4mM K_2_SO_4_, 249.5mM MgSO_4_, 0.8mM H_3_BO_3_, 0.125mM CuSO_4_, 0.06mM MnSO_4_, 1.7mM Fe(C_6_H_5_O_7_)] and an additional nitrate solution (428.5mM NH_4_NO_3_). Based on our previous experiments on the optimization of soil moisture, the control conditions (optimal water availability) were established when the soil moisture was maintained at a level of 12% of volumetric water content (VWC) in the soil, whereas the water deficit stress was achieved at 1.5% VWC. The soil moisture was measured manually using a time-domain reflectometer (TDR) EasyTest (Institute of Agrophysics, Polish Academy of Sciences, Poland.) The dielectric constant that was determined by the TDR provides an accurate measurement of soil water content and is essentially independent of soil texture, temperature, and salinity. The pre-prepared pots were moved to the greenhouse for 2 d. The pots were covered with foil to limit evaporation. Following this, germinated seeds were inserted evenly into pots (15 per pot) and grown in the greenhouse at 20/18 °C day/night, with a 16/8h photoperiod and 200 µE m^−2^ s^−1^ light intensity, which was provided by fluorescent lamps. During the control growth, the soil water content was maintained at 12% VWC by supplying water on daily basis. The water-stress treatment was performed as follows. Starting from 11 d after sowing (DAS) (reference point no. 1; Fig. 1) the moisture was gradually decreased by withholding irrigation and was controlled using TDR in order to achieve 3% VWC at 15 DAS (reference point no. 2). At this point, the second leaf had emerged, and the plants were transferred to a growth chamber with controlled conditions, where the temperature regime was set at 25/20 °C day/night, with a 16/8h photoperiod and 200 µEm^−2^s^−1^ light intensity. The soil moisture was maintained at 1.5% VWC. Water-stress treatment was applied for 10 d until 25 DAS (reference point no. 3). The experiment was set up with three replicates (pots) with each containing 15 plants for each genotype. At each of the above-indicated reference points, material from three independent plants (three biological replicates for leaves and roots) were collected for RNA extraction. Physiological analyses such as the leaf relative water content (RWC) and chlorophyll fluorescence were also performed. In summary, the water-stress experiment included three phases: (i) control growth (12% VWC), which lasted until 11 DAS; (ii) adaptation to the water deficit (3% VWC), which lasted 4 d; and (iii) water-deficiency stress (1.5% VWC), which lasted 10 d (15–25 DAS).

#### RWC analysis

RWC was calculated based on the formula RWC (%)=(FW – DW)/(TW – DW)×100, where: FW is the fresh weight of the detached second leaf, TW is the turgid weight of the second leaf, which was incubated in distilled water for 24h in darkness after detachment, and DW is the dry weight of the second leaf after it was dried in a dryer at 60 °C for 48h. Three plants from each of the three pots (as described above) were used for RWC analysis, which resulted in three biological replicates for each genotype and reference point (one biological replicate was represented by three plants from one pot).

#### Chlorophyll *a* fluorescence analysis

Chlorophyll *a* fluorescence was measured using a PocketPea fluorimeter (Hansatech, UK). Measurements of the second leaf of the three plants from each of the three pots as described above were taken. Before the measurements, the leaves were dark adapted for 30min and immediately afterwards were exposed to a pulse of saturating light at an intensity of 3500 µmol m^–2^ s^–1^ with a wavelength of 627nm. In the present studies, analysis of chlorophyll *a* fluorescence was focused on the curve of the electron transport between the O–J–I–P phases in which the O–J steps refer to the light reactions ([φPo/(1 – φPo)]) and the J–I–P steps refer to the biochemical reactions of photosystem II (PSII) [ψo/(1 – ψo)]. The efficiency of the light and biochemical reactions was calculated. The primary photochemistry of PSII was further evaluated using the following parameters: absorption flux (ABS), trapping flux (TR), electron-transport flux (ET), and dissipation flux (DI). The density of the active PSII reaction centres (RCs) per cross-section (CS), i.e. ABS/CS, TR_0_/CS, ET_0_/CS, DI_0_/CS, and RC/CS, were calculated using the phenomenological energy fluxes per excited CS (Strasser and Strasser, 1995; Strasser *et al.*, 2000). All of these parameters were tabulated during the water-deficiency experiment at the three reference points indicated above. The estimation of these parameters has been widely demonstrated to be associated with drought stress and plant vitality (Kalaji and Loboda, 2007; Chaves *et al.*, 2009; Kalaji *et al.*, 2011). Such measurements should allow the modular nature of PSII and any fluctuations in the efficiency of the light and dark reactions during drought stress to be understood.

#### RNA isolation

The dissected plant tissues were homogenized in a sterile, ice-cold mortar containing 500 µl of RLT buffer (RNeasy Plant Mini kit; Qiagen, Hilden, Germany). After homogenization, total RNA was extracted using an RNeasy Plant Mini kit according to the manufacturer’s instructions. RNA was concentrated from the column in 35 µl of sterile, RNase-free water by double elution. For the microarray analyses, RNA was additionally purified using precipitation in 1M lithium chloride, and each RNA precipitate was then dissolved in 15 µl of nuclease-free H_2_O. The yield and purity of the RNA was determined using a NanoDrop ND-1000 spectrophotometer (NanoDrop Technologies, Wilmington, USA). The integrity of the RNA was checked using an Agilent 2100 Bioanalyzer using an RNA 6000 Nano chip (Agilent Technologies, Santa Clara, USA).

#### Preparation of microarrays

The synthesis, labelling, and hybridization of cDNA and cRNA were carried out at the Genomics Core Facility, European Molecular Biology Laboratory (EMBL), Heidelberg, Germany. Total RNA was amplified using T7-based linear amplification and was Cy3 labelled in order to produce the target cRNA for use on the Agilent Barley Gene Expression Arrays. For each sample, 100ng of total RNA was used in the labelling reaction together with the spike-in (Agilent One Color Spike-in kit), which was diluted to obtain a final dilution of 1:10 000. All of the reagents for the labelling reaction were obtained from the Agilent Low Input Quick Amp kit. The labelled cRNA was purified using an RNeasy Mini kit (Qiagen) and quantified using a NanoDrop to obtain the yield values and specific activity values, which are related to the linear amplification and dye association, respectively. Samples with yields and specific activity greater than the recommended values for the 4×44K arrays were used for hybridization. A volume of 55 µl of 2× GE Hybridization mix (Agilent GE Hybridization kit) was added to the fragmented cRNA and 110 µl was loaded onto the Agilent 4×44k Barley Array within a gasket array slide sandwich. Arrays were hybridized in a rotisserie hybridization oven at 65 °C for 17h (Agilent p/n G2545a Hybridization Oven). After 17h, the slides were removed from the oven and washed with Agilent Wash Buffers containing Triton X. After the wash, the slides were scanned on an Agilent High Resolution Scanner using the green dye channel at a resolution of a 5 µm single pass scan with no extended dynamic range. The images were then analysed using the Agilent Feature Extraction software, version 10.7.3.1.

#### Microarray data analysis

The microarray data were analysed using GeneSpring GX 12.5 software (Agilent Technologies). Hybridization data from all of the biological replicates for each genotype were subjected to per chip normalization using the percentile shift method to the 75th percentile. A baseline transformation was then performed to the median of all of the samples. Statistical testing for differential expression was performed using either Student’s *t*-test or a two-way ANOVA followed by the Benjamini–Hochberg false discovery rate (FDR) correction for multiple testing (Benjamini and Hochberg, 1995). A gene was considered to be differentially expressed when the level of its expression in the mutant differed by at least three times in comparison with the parent variety [fold change (FC)≥3; *P≤*0.05 after FDR correction]. Raw microarray data, normalized intensity values, and corresponding metadata are accessible through the Gene Expression Omnibus (GEO) repository under accession number GSE73789.

#### Annotation of the Agilent Barley Gene Expression Microarray

The Agilent Barley Gene Expression Microarray (Agilent Technologies) was used for the global analysis of leaf and root transcriptomes of the WT cv. ‘Karat’ and the *rhl1.a* mutant. First, a *de novo* annotation of the array was done. Using a BLAST-based combinatorial strategy, 18 000 array probes were mapped to cDNAs that represented the 11 340 unique high-confidence (HC) genes that have been annotated in the barley genome. Taking into account that the estimated number of HC barley genes is 24 318 (International Barley Genome Sequencing Consortium, 2012), the Agilent Barley Gene Expression Microarray represents about 47% of barley genes. The main objective of the annotation of the Agilent Barley Gene Expression Microarray via the mapping of the array to the HC genes was to use their broad annotations provided by the PLAZA database (http://bioinformatics.psb.ugent.be/plaza/versions/plaza_v3_monocots; Proost *et al.*, 2015) which include Gene Ontology (GO) annotation, protein domains, homologous gene families, and the prediction of orthologous genes. This annotation of barley genes provided by PLAZA makes it the most comprehensive source of information, and we subsequently used it for all of the bioinformatic annotation of barley genes, including barley/Arabidopsis cross-species analysis.

#### Quantitative reverse transcription (RT)-qPCR

One microgram of total RNA was used in 20 µl reactions for cDNA synthesis using a Maxima First Strand cDNA Synthesis kit for RT-qPCR (Thermo Scientific). The cDNA that was obtained was then diluted 1:5 with ddH_2_O and used as a template for the quantitative PCR. All of the primers that were used in the qPCR were designed using Quant-Prime software (http://www.quantprime.de). The 10 µl qPCR contained 2 µl of diluted cDNA, 1 µl of the primer pair mixture (5 µM), and 5 µl of 2× Master Mix (LightCycler 480 SYBR Green I Master; Roche). The following qPCR protocol was used on a LightCycler 480 Real-Time PCR Instrument (Roche) using the SYBR Green I method: initial denaturation for 10min at 95 °C, followed by 45 cycles of 10s at 95 °C, 15s at 56 °C, and 10s at 72 °C, followed by a melting-curve analysis. The reference gene that was used in this study was *RPII* (*RNA polymerase II largest subunit*; EST: HO04I23S; Kwasniewski *et al.*, 2010). Data were analysed using LinRegPCR (Ramakers *et al.*, 2003) and Excel software (Microsoft). Calculations of the fold change of expression (FC) were made using the formula FC=*E* ^Δ*C*^_t_, where *E* is the mean value of the amplification efficiency of a given gene and Δ*C*_t_ corresponds to the difference between the mean *C*_t_ values of all of the biological replicates between the two samples that are being compared.

#### GO enrichment analysis

In order to identify the functional ontologies that are associated with the DEGs and to estimate the enrichment of the functional categories across the treatments, an enrichment analysis based on GO terms was performed using the PLAZA Monocots database version 3.0 (http://bioinformatics.psb.ugent.be/plaza/versions/plaza_v3_monocots; Proost *et al.*, 2015). The GO enrichment tool at PLAZA determines the over-representation of a certain GO term in a gene set compared with the genome-wide background frequency. The significance of over-representation was determined using the hypergeometric distribution followed by the Bonferroni method for multiple testing correction (corrected *P≤*0.01). Additionally, PLAZA was used to identify the corresponding barley orthologues in *Arabidopsis thaliana* taking into account the extensive annotation features that are available for this species.

## Results and discussion

### Effect of root hair phenotype on photosynthesis under water stress

**Fig. 2.**
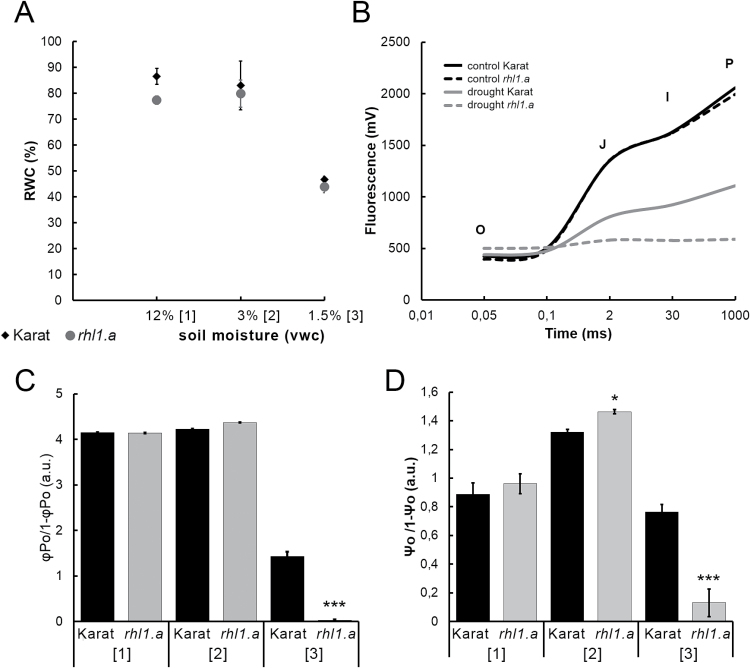
Physiological analyses of cv. ‘Karat’ and the *rhl1.a* mutant during the water-stress experiment. (A) Leaf RWC (%). According to a *t*-test, there were no significant differences between the genotypes at any time point (*P*≤0.05). (B) Transient chlorophyll *a* fluorescence induction curves under control conditions (12% VWC) and after 10 d of water stress (1.5% VWC). O, fluorescence intensity at 50 µs; J, fluorescence intensity at 2ms; I, fluorescence intensity at 30ms; P, maximum fluorescence. (C, D) Light reactions (φPo/1 – φPo) (C) and biochemical reactions (Ψo/1 – Ψo) (D) in PSII during the water-stress assay. The statistical differences between the WT ‘Karat’ and *rhl1.a* mutant are indicated for each time point (*P≤0.05, **P≤0.01, ***P≤0.001). a.u., arbitrary units. Numbers in square brackets represent the three time points of the experiment as shown in Fig. 1.

In order to gain an insight into the physiological changes in the ‘Karat’ and *rhl1.a* plants that were treated with water stress, a basic physiological assay, determining leaf RWC (%), was performed. RWC was analysed at the indicated time points, and the second leaf of a plant was always used as the object. It was clearly shown that a gradual decrease in soil moisture to 3% VWC did not influence the RWC parameter in either the WT ‘Karat’ or root-hairless *rhl1.a* mutant (Fig. 2A). The severe water stress lasting 10 d (1.5% VWC) resulted in a decrease in the RWC values to 40–50% in both of the genotypes studied.

In addition to the RWC, a more detailed analysis of the photosynthetic apparatus condition was performed using the O–J–I–P test (Govindjee, 2004). Although both of the genotypes that were studied, the *rhl1.a* mutant and its WT parent, displayed changes in the performance of the photosynthetic apparatus under water stress, the reaction of the *rhl1.a* mutant was much stronger. The chlorophyll *a* fluorescence induction curves were determined under control conditions and after water stress for both genotypes (Fig. 2B). The shape of the O–J–I–P fluorescence rise curve is related to a change in the photosynthetic electron transport. Under normal conditions (12% VWC, reference point 1), there were no differences between the *rhl1.a* mutant and its WT parent with regard to chlorophyll *a* fluorescence. After 10 d of severe water stress (1.5%, VWC, reference point 3), a flattening of the curve was observed for both genotypes. This indicated that the rate of the reduction of quinone-type acceptors during the early photochemistry reactions was significantly affected by water stress. A much slower fluorescence rise and significantly lower ‘P’ value (maximal value of fluorescence) were observed for the ‘Karat’ plants after water-stress treatment compared with the control conditions. On the other hand, the curve of the electron transient between the O, J, I, and P phases in the case of the *rhl1.a* mutant after water-stress treatment was totally flat without any O, J, I, and P peaks (Fig. 2B). These observations indicated a possible reduction or even inhibition of light and biochemical reactions in the *rhl1.a* mutant. The analysis of the efficiency of light reactions [φPo/(1 – φPo); Fig. 2C] and biochemical reactions [ψo/(1 – ψo); Fig. 2D] in PSII showed that both parameters were markedly affected in both genotypes; however, much more pronounced and dramatic changes were noted in *rhl1.a.* The exposure to water stress decreased the efficiency of light reactions and biochemical reactions that are performed in PSII to almost zero in the mutant Fig. 2C, D.

In order to evaluate further the effect of water stress on the primary photochemistry of PSII, we examined ABS, TR, ET, DI, and the density of active PSII RCs per excited CS, i.e. ABS/CS, TR_0_/CS, ET_0_/CS, DI_0_/CS, and RC/CS (Table 1). The absorption of photons by the chlorophyll molecules in the antenna complex (ABS/CS) and trapped energy (TR_0_/CS) fluxes decreased significantly to 41 and 31% of the control in the ‘Karat’ plants and to 7 and 2% of the control in the *rhl1.a* mutant after 10 d of severe water stress, respectively. Both genotypes were also strongly affected by water stress with regard to the inhibition of electron transport in a PSII CS (ET_0_/CS). However, the values for the *rhl1.a* mutant were once again close to zero (0.39% of control), whereas in the case of the WT ‘Karat’, the reduction was at the level of 30% of the control. This parameter was in strong correlation with the number of active RCs (RC/CS). A decrease to 30 and 1.4% of the control in the ‘Karat’ and *rhl1.a* mutant, respectively, was observed in the number of active RCs.

**Table 1.** Energy fluxes per excited CS in the WT cv. ‘Karat’ and rhl1.a mutant during the drought assay

**VWC (%**)	**Genotype**	**ABS/CS (%**)	***P***	**DI**_**0**_ **/CS (%**)	***P***	**TR**_**0**_ **/CS (%**)	***P***	**ET**_**0**_ **/CS (%**)	***P***	**RC/CS (%**)	***P***
12	‘Karat’	100		100		100		100		100	
	*rhl1.a*	100	NS	100	NS	100	NS	100	NS	100	NS
3	‘Karat’	91		90		91		110		113	
	*rhl1.a*	104	**	99	**	105	**	132	***	128	*
1.5	‘Karat’	41		80		31		30		30	
	*rhl1.a*	7	***	26	**	2	***	0.39	***	1.4	***

ABS/CS, absorption flux per CS; TR_0_/CS, trapped energy flux per CS; ET_0_/CS, electron-transport flux per CS; DI_0_/CS, dissipated energy flux per CS; RC/CS, reaction centres per CS. Values in the table are presented as a relative values (% of the control). Statistical significance was evaluated with Student’s *t*-test between ‘Karat’ and the *rhl1.a* mutant at each time point (*P≤0.05, **P≤0.01, ***P≤0.001, *n*=3). NS, not significant.

### Transcriptomic response of the root-hairless mutant and its parent cultivar under water-stress conditions

As presented above, the root hair phenotype influenced photosynthesis under water stress, and this indicates that the root hairs help to maintain plant function under water-deficiency conditions. In order to investigate the molecular mechanisms that are potentially involved in the root hair phenotype-related response to harmful environmental conditions, a series of transcriptomic analyses were carried out by the comparative analysis of the *rhl1.a* mutant and its parent ‘Karat’.

#### General characteristics of the root hair formation transcriptome

**Fig. 3.**
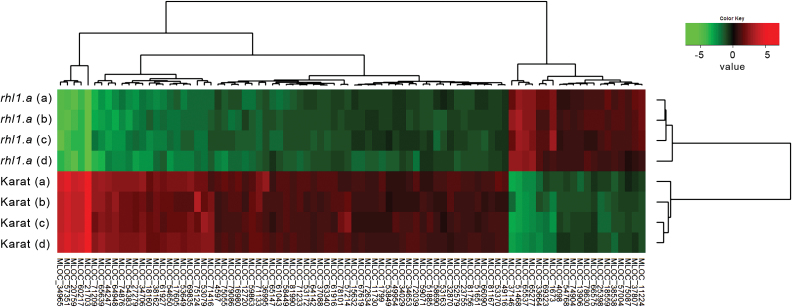
Hierarchical clustering of 86 genes that were differentially expressed in the root tip of the WT cv. ‘Karat’ versus its root-hairless mutant *rhl1.a.* (*P≤*0.05 after FDR correction; FC≥3). Letters in parentheses (a–d) represents four biological replicates of each genotype.

As the first step towards a characterization of the transcriptomic differences in the WT and root-hairless plants response to water stress, the transcriptomic differences in the root hair formation zone of plants that had been grown in control, sterile, aeroponic conditions were analysed using the identical experimental set-up that we had used previously with the Affymetrix GeneChip Barley1 Genome Array (Kwasniewski *et al.*, 2010). The differential analysis of transcriptomes from 1cm long root tip fragments of the WT ‘Karat’ and its root-hairless mutant *rhl1.a* resulted in the identification of 66 down-regulated and 20 up-regulated HC genes in the roots of *rhl1.a* in comparison with the WT parent (FC≥3; *P*≤0.05 after FDR correction; Fig. 3; Supplementary Table S1 at *JXB* online). Functional annotation of the differentially regulated genes, which was carried out using the PLAZA tools and the iRootHair database as references (http://www.iroothair.org; Kwasniewski *et al.*, 2013a), permitted the identification of the putative orthologues of four genes that are involved in root hair morphogenesis in *A. thaliana*. The genes encoded RHD4 (ROOT HAIR DEFECTIVE4), a phosphatidylinositol-4-phosphate phosphatase that is required for root hair tip growth; COW1 (CAN OF WORMS1), a phosphatidylinositol transfer protein that is also essential for root hair tip growth; SHV3 (SHAVEN 3), a glycerophosphoryl diester phosphodiesterase-like protein that is involved in cell wall cellulose accumulation and pectin linking; and XTH26, which is a xyloglucan endotransglucosylase/hydrolase 26 that is involved in cell wall metabolism. Additionally, the *HvEXPB1* gene-encoding expansin B protein, which is responsible for cell wall loosening during the initiation of root hair growth (Kwasniewski and Szarejko, 2006), was found in the current analysis (see Supplementary Table S1). Interestingly, *HvEXPB* was not detectable on the Affymetrix GeneChip Barley1 Genome Array as its ESTs were identified after the design of the Affymetrix GeneChip.

In order to test the reliability of the results that were obtained by the Agilent Barley Gene Expression Microarray, the expression of DEGs was validated using sensitive RT-qPCR. The analysis of 20 genes that were selected based on their expression, which covered a wide range of microarray expression values, successfully validated the data obtained by the Agilent Barley Gene Expression Microarrays (see Supplementary Fig. S1). Therefore, the preliminary experiment that was carried out using a simple model of root hair transcriptome (*rhl1.a* vs WT ‘Karat’) that had been used previously revealed that the Agilent Barley Gene Expression Microarray system, supported by the recent *de novo* annotation, can be used successfully for transcriptome profiling in barley.

#### Comparative analysis of the drought-induced transcriptomes of *rhl1.a* and its parent ‘Karat’

**Fig. 4.**
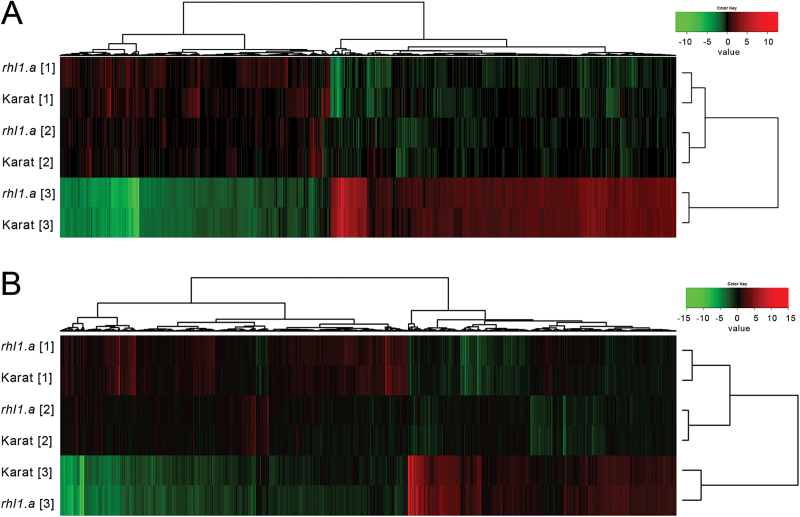
Hierarchical clustering of genes that were differentially expressed during the water-stress experiment in the roots (A, 4706 genes) and leaves (B, 7434 genes) of the cv. ‘Karat’ and its root-hairless mutant *rhl1.a* (*P≤*0.05 after FDR correction; FC≥3). Numbers in square brackets represent the three time points of the experiment as shown in Fig. 1.

Our model system of a root-hairless mutant *rhl1.a* and its WT parent cultivar ‘Karat’ was used to investigate the molecular mechanisms that are potentially involved in the root hair phenotype-related response to water-stress conditions. A time-course water-stress experiment was applied to both genotypes, and transcriptome analyses were performed in root and leaf tissues at three reference points: (1) normal conditions, soil moisture 12%; (2) the onset of water stress, soil moisture 3%; and (3) severe stress treatment, soil moisture 1.5% (Fig. 1). Microarray data analysis was performed as a calculation of the differential expression of genes within a genotype between subsequent reference points (2 vs 1 and 3 vs 1). Global transcriptome analysis, which was carried out in the roots and leaves of both genotypes during water stress, revealed that the roots and leaves differed in the expression of stress-related, DEGs. More water-stress-related DEGs were expressed in the leaves than in the roots at each stage of water-stress treatment (Fig. 4). Specifically, 4706 genes in total were differentially expressed in roots during two subsequent stages of drought stress (reference points 2 vs 1 and 3 vs 1), whereas 7434 were detected as differentially expressed in leaves under the same conditions (Fig. 4; *P*≤0.05 after FDR correction; FC≥3). Hierarchical clustering revealed that the changes that occurred during the onset of stress were less prominent in roots than in leaves (differences between time points 1 and 2; Fig. 4), whereas 10 d of the severe stress treatment influenced both the root and leaves transcriptomes harshly (differences between clustered time points 1 and 2 and time point 3; Fig. 4).

**Fig. 5.**
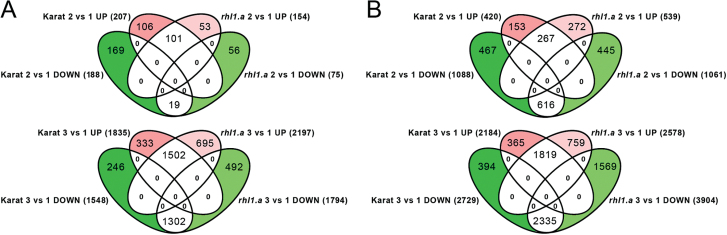
Comparative analysis of the numbers of DEGs in the roots (A) and leaves (B) of the WT cv. ‘Karat’ and its root-hairless mutant *rhl1.a* during subsequent stages of the water-stress experiment (*P≤*0.05 after FDR correction; FC≥3). Coloured parts of the Venn diagrams represent the ‘Karat’-specific or *rhl1.a*-specific subsets of genes that were up-regulated (red) or down-regulated (green) during the subsequent stages of the experiment in comparison with the control conditions: 2 vs 1, genes up- or down-regulated during the onset of water stress; 3 vs 1, genes up- or down-regulated after 10 d of severe water stress.

The general response of the ‘Karat’ transcriptome to stress conditions was similar to the response of the *rhl1.a* transcriptome, as their expression profiles always clustered together within the time points that were examined, in both roots and leaves (Fig. 4). However, a detailed analysis of the lists of genes that were regulated by water stress in subsequent stages of the experiment revealed distinct differences in the stress response between the WT and the *rhl1.a* mutant. First, twice as many genes responded to the rapid decrease in soil moisture (time point 2 vs 1) in the roots of the WT (395 genes) than in the *rhl1.a* mutant (229 genes) (Fig. 5; *P*≤0.05 after FDR correction; FC≥3). The same conditions (soil moisture 3% VWC) affected the leaf transcriptome more radically than the root transcriptome, although there were no quantitative differences in the response of the leaf transcriptome between the WT and *rhl1.a* (1508 vs 1600 DEGs in ‘Karat’ and in the *rhl1.a* mutant, respectively). It was possible to distinguish the genotype-specific subsets of genes that were regulated by water stress in both organs (coloured parts of the Venn diagrams in Fig. 5), as well as the common sets of genes that responded similarly in both genotypes.

**Fig. 6.**
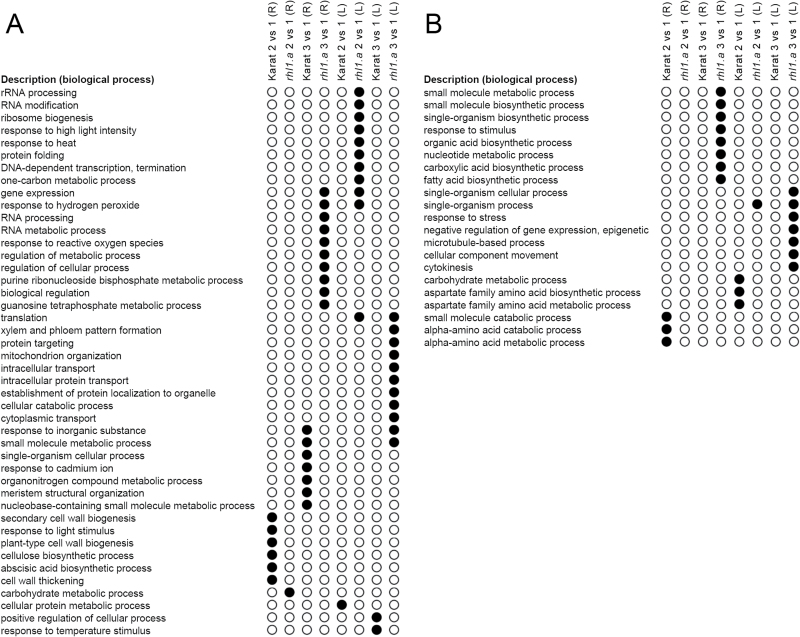
GO categories (Biological Processes) that were over-represented in the ‘Karat’-specific or *rhl1.a*-specific subsets of genes: up-regulated (A) or down-regulated (B) genes during subsequent stages of the experiment in comparison with the control conditions in the roots (R) or leaves (L) of both genotypes (corrected *P*<0.01).

The severe water stress that was maintained for 10 d (time point 3 vs 1) affected the transcriptomes of roots and leaves drastically. Here, however, many more genes were deregulated in the roots and leaves of the *rhl1.a* mutant than in the WT (3991 vs 3383 in roots, and 6482 vs 4913 in leaves of the *rhl1.a* mutant and ‘Karat’, respectively; Fig. 6).

#### Functional annotation of root hair phenotype-specific subsets of water-stress-regulated genes

Genotype-specific, and therefore root hair phenotype-specific, subsets of water-stress-regulated genes were analysed using the GO enrichment approach in which the enrichment of the functional annotations (GOs) of barley genes was performed using the PLAZA Monocots 3.0 database. Thus, the subsets of the genotype-specific genes that were differentially expressed at the onset of water stress (time point 2 vs 1) and after 10 d of severe stress (time point 3 vs 1) in the roots and leaves of both genotypes were analysed (coloured parts of the Venn diagrams in Fig. 5). The analysis resulted in the identification of over-represented Biological Processes (corrected *P*<0.01), which were up- or down-regulated specifically in the roots and leaves of either WT ‘Karat’ or its root-hairless mutant *rhl1.a* (Fig. 6, Supplementary Table S2 at *JXB* online).

##### Differences between ‘Karat’ and the *rhl1.a* mutant at the onset of water stress

At the onset of water stress, more genes responded to the rapid decrease in soil moisture in the roots of the WT than in the roots of the *rhl1.a* mutant. Among these, 106 genes were specifically up-regulated in the roots of ‘Karat’ but not in the roots of the hairless mutant (Fig. 5; *P*≤0.05 after FDR correction; FC≥3). Their GO enrichment analysis revealed that these genes represented six significantly over-represented (corrected *P*<0.01) Biological Processes (BPs), including the ABA biosynthetic process, the cellulose biosynthetic process, cell wall thickening, plant-type cell wall biogenesis, secondary cell wall biogenesis, and the response to light stimulus (Fig. 6). Detailed analysis revealed that two barley genes, MLOC_18300 and MLOC_43893 (BP: ABA biosynthetic process), which were highly up-regulated in the roots of the WT at the beginning of water stress, encode enzymes that belong to 9-*cis*-epoxycarotenoid dioxygenase (NCED, Table 2). In Arabidopsis, NCED is a key enzyme involved in ABA biosynthesis that cleaves 9-*cis* xanthophylls to xanthoxin, which is a precursor of ABA (Tan *et al.*, 2003). Its expression is stimulated by water deprivation, and the stress-inducible ABA biosynthesis is associated with an increased expression of *NCED* genes in both leaf and root tissues (Thompson *et al.* 2000). Interestingly, recent studies have revealed that a specific allele of the 9-*cis*-epoxycarotenoid dioxygenase gene is preferentially selected in elite varieties of rice. Its functional analysis indicates that, in upland rice, this *NCED* allele probably confers a greater drought resistance by raising endogenous ABA levels, and additionally is associated with a higher density of lateral roots, thus suggesting its involvement in upland rice fitness (Lyu *et al.*, 2013). Therefore, the specific, strong up-regulation of two *NCED* genes in the roots of the WT cv. ‘Karat’ indicated that the WT plants, which produce normal root hairs, respond to water stress with a more efficient stimulation of ABA biosynthesis than the root-hairless mutant. This, consequently, could result in an earlier or more efficient induction of an ABA-related systemic response leading to adaptation to the stress conditions.

**Table 2.** Selected genes differentially expressed in roots of the ‘Karat’ and the rhl1.a mutant at the onset of water stress

Regulation	MLOC *H. vulgare*	Putative orthologue in *A. thaliana*	Function in *A. thaliana*	Expression in water-stress experiment					
				‘Karat’			*rhl1.a*		
				1	2	3	1	2	3
Up-regulated at the onset of water stress in roots of ‘Karat’	MLOC_18300	9-*cis*-Epoxycarotenoid dioxygenase (NCED)	Key enzyme involved in the ABA biosynthesis	–1.037	0.589	1.987	–1.292	–0.284	3.081
	MLOC_43893	9-*cis*-Epoxycarotenoid dioxygenase (NCED)	Key enzyme involved in the ABA biosynthesis	–2.098	0.296	3.494	–2.421	–0.937	3.665
	MLOC_66568	*CELLULOSE SYNTHASE A4* (*CESA4*, AT5G44030),	Cell wall biogenesis; expression controlled by NST2 and SND1	0.346	2.039	–1.507	–0.192	0.748	–1.310
	MLOC_68431	*CELLULOSE SYNTHASE A7* (*CESA7*, AT5G17420),	Cell wall biogenesis; expression controlled by NST2 and SND1	0.368	2.050	–3.230	–0.394	0.929	–4.023
	MLOC_43749	*CELLULOSE SYNTHASE A8* (*CESA8*, AT4G18780),	Cell wall biogenesis; expression controlled by NST2 and SND1	0.495	2.338	–3.836	–0.289	0.699	–3.960
	MLOC_75040	*COBRA-LIKE4* (*COBL4*, AT5G15630)	Cell wall biogenesis; expression controlled by NST2 and SND1	–0.258	1.632	–2.414	0.128	0.494	–2.701
	MLOC_63741	*KORRIGAN 1* (*KOR1*, At5g49720)	Cell wall biogenesis; expression controlled by NST2 and SND1	0.149	2.107	–0.670	–0.051	1.102	–1.012
	MLOC_5406	*FLOWERING LOCUS T* (*FT*, AT1G65480)	Control of stomata aperture; drought- escape response	–1.889	0.721	2.052	–1.898	–1.431	1.223
Up-regulated in roots of *rhl1.a* mutant	MLOC_65860	*CHLOROPLAST PROTEIN 12* (*CP12-1*, At2g47400)	Regulation of Calvin cycle enzymes GAPDH and PRK	–2.540	–1.043	2.069	–2.419	0.422	2.562
	MLOC_58999	*PHOSPHORIBULOKINASE* (*PRK*, AT1G32060)	Calvin cycle enzyme regulated by CP12 protein	–0.427	–0.877	2.055	–0.928	0.764	3.272

1, 2, 3 - Subsequent time points of the water-stress experiment. The strength of expression is indicated by shading (low–high: green–yellow–orange–red).

The BPs that are related to cellulose biosynthesis and cell wall biogenesis were represented by seven genes (Table 2). Five of them: MLOC_66568, MLOC_68431, MLOC_43749, MLOC_75040, and MLOC_63741, are orthologues of the Arabidopsis genes *CELLULOSE SYNTHASE A4* (*CESA4*, AT5G44030), *CELLULOSE SYNTHASE A7* (*CESA7*, AT5G17420), *CELLULOSE SYNTHASE A8* (*CESA8*, AT4G18780), *COBRA-LIKE4* (*COBL4*, AT5G15630), and *KORRIGAN 1* (*KOR1*, At5g49720), respectively. Interestingly, the recent work of Taylor-Teeples *et al.* (2015) demonstrates that all of these genes are involved in cell wall biogenesis in Arabidopsis and belong to the same regulatory network. Their expression is tightly controlled by NAC-domain binding transcription factors, NST2 and SND1, which directly bind to their promoters via a specific, conserved *cis*-regulatory element. The expression of all of these genes in barley was specifically strongly up-regulated in the roots of the WT plants at the onset of water stress. This orchestrated response suggests that the MLOC_66568, MLOC_68431, MLOC_43749, MLOC_75040, and MLOC_63741 genes belong to a single regulatory network in barley, which is efficiently activated in roots at an early phase of water stress in the WT plants but not in the root-hairless mutant. It has already been demonstrated that the root growth of seedlings that are subjected to mild water stress is often less inhibited than the shoot growth, and this has been considered to be a mechanism of the adaptation of plants to drought conditions (Sharp and Davies, 1989). This different reaction of roots and shoots can be explained by water-stress-induced changes in the cell wall composition and the action of cell wall-loosening molecules, such as expansins or xyloglucan endotransglycosylases (Tenhaken, 2014). Moreover, the efficiency of the synthesis and the incorporation of newly synthesized polysaccharides into the walls of root cells could influence the water-stress adaptation potential in plants. It was shown, for example, that the efficiency of the biosynthesis of cell wall polysaccharides such as pectins, hemicelluloses, and α-cellulose during water stress was quantitatively higher in the roots of drought-tolerant than in drought-sensitive wheat cultivars (Piro *et al.*, 2003). Taking this data into account, it could be suggested that the efficient induction of cell wall biosynthesis at the onset of water stress in the WT plants (but not in the root-hairless mutant) could result in a better adaptation to stress conditions. It is worth noting that the severe drought (time point 3 vs 1) resulted in a drastic down-regulation of all of the above-mentioned cellulose biosynthesis and cell wall biogenesis genes, and that this is in line with the observation of the cessation of root growth under severe drought conditions (Opitz *et al.*, 2014).

A particularly interesting observation of the specific response of the roots of the WT plants involves the expression of the gene MLOC_5406 (BP: response to light stimulus), which encodes a PEBP (phosphatidylethanolamine-binding protein) family protein. The expression of this gene is specifically activated in the roots of the WT at the onset of water stress but not in the roots of the *rhl1.a* mutant. MLOC_5406 is a putative orthologue of the florigen gene *FLOWERING LOCUS T* (*FT*) in Arabidopsis (AT1G65480), which is involved in the control of the stomata aperture in response to a photoperiod (Ando *et al.*, 2013). Recent studies have revealed existing relationships between drought stress and photoperiod in the activation of the florigen genes, including *FT*. Here, *FT* plays a role as a key factor for a drought-escape response that is facilitated by ABA (Riboni *et al.*, 2013). The authors suggested that the drought episodes that occur in spring may be a signal to plants of the possible more severe drought conditions to follow in the summertime, and thus the activation of a drought-escape response might be advantageous. Therefore, based on our data, it can be postulated that WT plants activate the expression of *FT* orthologous gene as an early response to water stress, presumably as a component of a drought-escape adaptive mechanism. However, the root-hairless mutant was not able to sense the stress efficiently and/or its response to water deprivation was not as effective as it was in the WT.

In comparison with the WT cv. ‘Karat’, at the onset of water stress significantly fewer genes were specifically up-regulated in the roots of the root-hairless mutant *rhl1.a* (53 genes; Fig. 5; *P*≤0.05 after FDR correction; FC≥3). GO enrichment analysis led to the identification of a single over-represented GO category (BP: carbohydrate metabolic process; corrected *P*<0.01; Fig. 6). Two genes that represented this category, MLOC_65860 and MLOC_58999, are putative orthologues of Arabidopsis genes At2g47400 and AT1G32060, respectively, and are involved in the Calvin cycle. At2g47400 encodes a protein that belongs to the chloroplast protein CP12 family, which has been shown to regulate the activity of two Calvin cycle enzymes, glyceraldehyde-3-phosphate dehydrogenase (GAPDH) and phosphoribulokinase (PRK), which is encoded by AT1G32060. Interestingly, At2g47400 is a *CP12-1* gene whose expression in Arabidopsis is not restricted to the above-ground organs but is also specifically expressed in root tips and lateral roots (Singh *et al.*, 2008). Therefore, the authors suggested that the redox-sensitive CP12 proteins may have a wider role in non-photosynthetic plastids.

The up-regulation of two tightly related genes that are involved in the same Calvin cycle process at the onset of water stress in the roots of the *rhl1.a* mutant but not in the WT suggests their putative role in response to stress conditions. The increased expression of MLOC_65860 and MLOC_58999 in the *rhl1.a* mutant might lead to the up-regulation—in comparison with WT—of the light-independent photosynthetic processes. Interestingly, at the onset of drought, significant differences (*P*≤0.05) were observed in the biochemical reaction efficiencies between the WT and *rhl1.a* mutant (Fig. 2D), where the *rhl1.a* mutant seemed to be more efficient. Taking into account the results of the specific up-regulation of ABA-related signalling processes in the roots of the WT that have already been discussed, these differences in biochemical reactions could result from (i) the earlier response to water stress in the WT, for example, via the ABA-dependent pathway; (ii) the specific up-regulation of Calvin cycle-related enzymes in the roots of the mutant with unknown consequences for the leaves; or (iii) both.

Our hypothesis about the less efficient activation of stress-preventing mechanisms in the root-hairless mutant can be supported by the results of the GO enrichment analysis of 272 genes, which were specifically up-regulated in the leaves of the mutant but not in the WT plants (Fig 6B). The analysis revealed the significant up-regulation of the genes that are involved in the response to abiotic stimuli, including the response to hydrogen peroxide, heat, and high light intensity, as well as the genes involved in the regulation of gene expression, rRNA processing, ribosome biogenesis, and protein stabilization (Fig. 6A; Supplementary Table S2). More specifically, this gene set was highly enriched in numerous heat-shock proteins, ribosomal proteins, and stress-activated transcription factors, which are genes that are related to later stages of the stress response.

##### Consequences of prolonged water stress on the WT and *rhl1.a* transcriptomes

An extended period of severe stress caused more drastic transcriptome changes in the roots and leaves of the *rhl1.a* mutant than in those of the WT. Expression of about 1200 genes was specifically affected by a prolonged water stress in the mutant, whereas about 600 were specifically regulated in the WT (coloured parts of the Venn diagrams in Fig. 5). In the roots of the mutant, the largest number of specifically up-regulated genes constituted those related to the response to abiotic stimuli and involved in stress-stimulated transcription regulation (Fig. 6A; Supplementary Table S2). On the other hand, a profound down-regulation of various biosynthetic processes was observed specifically in the roots of the root-hairless mutant but not in the WT plants (Fig. 6B; Supplementary Table S2).

Even more drastic genotype-specific differences in the severe stress response were observed in the leaves, where 6482 genes responded to stress in the mutant and 4913 in the WT. In the *rhl1.a* mutant, expression of more than 2300 genes was specifically affected by a prolonged water deficiency (*P*≤0.05 after FDR correction; FC≥3) whereas in the ‘Karat’ cultivar, about 750 genes were specifically regulated (coloured parts of the Venn diagrams in Fig. 5). It is worth noting that the majority of genes specifically affected by the prolonged water stress in the leaves of the mutant were down-regulated (1569 genes). They were involved mostly in cytokinesis- and microtubule-based process, including cellular component movement, the response to stress, and epigenetic regulation (Fig. 6B; Supplementary Table S2). The genes that were specifically up-regulated by severe drought in the leaves of the mutant, represented cellular catabolic processes, regulation of translation and maintenance of protein stability, cytoplasmic transport, the response to inorganic substances, and mitochondrion organization (Fig. 6A). Based on the results of comparative transcriptome analysis between the WT and *rhl1.a*, we concluded that drastic misregulation of the *rhl1.a* leaf transcriptome could explain a much stronger damage to PSII in the *rhl1.a* mutant than in its parent cultivar observed after 10 d of the water stress (Fig. 2C, D).

#### Putative stress sensing and signalling features of the root hair formation transcriptome

The preliminary analysis of the transcriptome of the root hair formation zone in the WT ‘Karat’ and its root-hairless mutant *rhl1.a* allowed the genes whose expression was affected in the mutant in comparison with the WT parent to be identified (Fig. 3). A detailed analysis resulted in the identification of the genes whose function was potentially related to sensing and signalling water stress (Table 3). Interestingly, the main category of these genes encoded proteins related to the Rho-like GTPases of plants (ROPs), which serve as signalling switches that control a wide variety of cellular processes, including cell morphogenesis, cell division, and cell differentiation, but that also are involved in ABA signalling. The barley gene MLOC_53849, which was down-regulated in the root hair transcriptome of the root-hairless mutant, is an orthologue of the *RHO OF PLANTS 11* (*ROP11*) gene in Arabidopsis. *ROP11* acts in an early step in ABA signalling as a negative regulator by protecting ABI1 phosphatase activity from being inhibited by the ABA receptor RCAR1/PYL9 (Li and Liu, 2012; Li *et al.*, 2012). Two other genes, MLOC_53172 and MLOC_57351, which are also down-regulated in the root hair transcriptome of the *rhl1.a* mutant, are orthologues of the Arabidopsis genes *ROP GUANINE NUCLEOTIDE EXCHANGE FACTOR 2* (*ROPGEF2*) and *ROP GUANINE NUCLEOTIDE EXCHANGE FACTOR 3* (*ROPGEF3*), respectively. These genes encode members of the ROPGEF (guanine nucleotide exchange factor) family, which contains the PRONE domain (plant-specific Rop nucleotide exchanger) that interacts exclusively with members of the ROP subfamily. It was shown that ROPGEF2 interacts strongly with ROP11, and it is postulated that ROPGEF2 might be responsible for the activation of ROP11 in vascular tissue (Li and Liu, 2012). Interestingly, it was also shown that another member of ROPGEF in Arabidopsis, ROPGEF4, acts as a functional regulator of ROP11 GTPase in the process of ABA-mediated stomatal closure (Li and Liu, 2012).

**Table 3.** Root hair transcriptome genes that have a potential role in drought sensing and signalling

Category	MLOC in *H. vulgare*	Putative orthologue in *A. thaliana*	Function	Reference(s)	Expression in roots in aeroponic conditions	
					‘Karat’	*rhl1.a*
ABA signalling	MLOC_53849	AT5G62880	*RHO OF PLANTS 11* (*ROP11*) is a member of the ROP GTPase gene family. ROP11 is specifically regulated by ROPGEF1 and ROPGEF4 and functions in ABA-mediated stomatal closure. ROP11 acts in an early stage of ABA signalling and is its negative regulator by protecting ABI1 phosphatase activity from inhibition by the ABA receptor RCAR1/PYL9.	Li and Liu (2012); Li *et al.* (2012)	0.756	–0.902
ABA signalling	MLOC_53172	AT1G01700	*ROP* (*RHO OF PLANTS*) *GUANINE NUCLEOTIDE EXCHANGE FACTOR 2* (*ROPGEF2*) encodes a member of the KPP-like gene family (kinase partner protein), as well as being a member of the RopGEF (guanine nucleotide exchange factor) family that contains the novel PRONE domain (plant-specific Rop nucleotide exchanger), which is active exclusively towards members of the Rop subfamily. ROPGEF2 may be responsible for ROP11 activation in vascular tissue.	Li and Liu (2012)	1.269	–1.205
ABA signalling	MLOC_57351	AT4G00460	*ROP* (*RHO OF PLANTS*) *GUANINE NUCLEOTIDE EXCHANGE FACTOR 3* (*ROPGEF3*) encodes a member of the KPP-like gene family (kinase partner protein), as well as being a member of the RopGEF (guanine nucleotide exchange factor) family that contains the novel PRONE domain (plant-specific Rop nucleotide exchanger), which is active exclusively towards members of the Rop subfamily.	-	4.580	–4.523
ABA signalling	MLOC_74876	AT4G24580	*ROP1 ENHANCER 1* (*REN1*) encodes a Rho GTPase activation protein (RhoGAP) that has a PH domain. REN1 is a Rho GTPase-activating protein that interacts with ROP1 (a Rho GTPase) and regulates pollen tube development. REN1 is required for restricting the ROP1 activity to the pollen tube tip, thus controlling growth polarity. Moreover, REN1 was found to be among 200 of the core root epidermal genes that may potentially regulate in the differentiation of root epidermis cells in Arabidopsis.	Bruex *et al.* (2012); Hwang *et al.* (2008)	2.144	–2.373
Calcium signalling	MLOC_54650	AT5G12380	Annexin 8 (ANNAT8) functions in calcium ion binding and calcium-dependent phospholipid binding. Annexins transduce calcium signals into adaptive responses and are activated in cells or tissues that have been exposed to osmotic stress, ABA, or water deficiency.	Kovács *et al*. (1998); Lee *et al.* (2004); Cantero *et al.* (2006)	2.021	–2.006
Calcium signalling	MLOC_58690	AT5G37770	*CALMODULIN-LIKE 24* (*ATCML24*) encodes an EF-hand domain that contains a calcium-binding protein. ATCML24 is a potential Ca^2+^ sensor that may enable responses to ABA, day length, and the presence of various salts. It is suggested that CML24 may act downstream of ABA perception, thus mediating cellular responses to ABA-induced Ca^2+^ fluctuations. CML24 expression is highly responsive to diverse environmental and hormonal stimuli. Moreover, it regulates pollen tube growth by modulating the actin cytoskeleton and controlling the concentration of cytosolic Ca^2+^.	Delk *et al.* (2005)	0.972	–1.145
ROS signalling	MLOC_15632	AT5G14130	Encodes a peroxidase that is involved in the response to oxidative stress; its expression is up-regulated by auxin that is applied externally (indole-3-acetic acid).	Goda *et al.* (2004)	0.921	–0.984
ROS signalling	MLOC_49954	AT5G05340	*PEROXIDASE 52* (*AtPrx52*) contains an ABA response element in its promoter and is up-regulated by ABA treatment but is down-regulated in *rpk1* mutant; it is up-regulated in roots in response to phosphate deprivation.	Osakabe *et al.* (2005); Misson *et al.* (2005)	0.996	–0.948
ROS signalling	JQ649324.1	-	*PEROXIDASE 2* (*PRX2*) encodes a root hair-specific class III peroxidase involved in ROS formation. PRX2 may mediate the root hair initiation process in a ROS- dependent manner.	Kwasniewski *et al.* (2013b)	Strongly down-regulated in *rhl1.a*	
ROS signalling	JQ649323.1	-	*PEROXIDASE 45* (*PRX45*) encodes a root hair-specific class III peroxidase that is involved in ROS formation. PRX45 may mediate the root hair initiation process in a ROS-dependent manner.	Kwasniewski *et al.* (2013b)	Strongly down-regulated in *rhl1.a*	

The strength of expression is indicated by shading (low–high: green–yellow–orange–red).

The fourth ROP-related gene that was found during the differential gene expression analysis of the root hair formation zone in the *rhl1.a* mutant and WT was MLOC_74876, which is an orthologue of *ROP1 ENHANCER 1* (*REN1*) in Arabidopsis, which encodes a Rho GTPase activation protein (RhoGAP) with a PH domain. REN1 interacts with ROP1 and regulates pollen tube tip growth by the total inhibition of Rho GTPase at the cell apex (Hwang *et al.*, 2008). To date, its role in regulating root hair development is not known; however, microarray data analysis has revealed its expression in trichoblasts. Moreover, *REN1* is down-regulated in the roots of the *rhd6* (*root hair defective 6*) mutant in Arabidopsis (Bruex *et al.*, 2012), in a similar manner to its orthologue MLOC_74876 in the *rhl1.a* mutant in barley.

The second category of the putative stress sensing and signalling components of the root hair formation transcriptome in barley constitutes the genes that are involved in calcium signalling. The barley gene MLOC_54650, which is down-regulated in the root-hairless mutant, is an orthologue of *ANNEXIN 8* (*ANNAT8*) in Arabidopsis. Plant annexins are calcium-binding proteins that transduce calcium signals into adaptive responses, and that are activated in cells or tissues that are exposed to osmotic stress, ABA, or water deficiency (Kovács *et al.*, 1998; Lee *et al.*, 2004). Interestingly, *ANNAT8* showed the highest transcript abundance among all annexin genes in Arabidopsis under dehydration and NaCl stress (Cantero *et al.*, 2006). Another gene that belongs to the calcium signal transducers that is differentially expressed in the *rhl1.a* mutant under water-deficiency treatment is MLOC_58690, which is an orthologue of *CALMODULIN-LIKE 24* (*ATCML24*) in Arabidopsis. *ATCML24* encodes an EF-domain-containing calcium-binding protein whose expression occurs in all of the major organs, especially in regions that are undergoing growth (Delk *et al.*, 2005). *ATCML24* transcript levels increase in plants that are subjected to stress, including hydrogen peroxide, ABA, and salt treatment. Transgenic Arabidopsis lines that have a reduced level of *CML24* expression are resistant to the ABA-induced inhibition of germination and seedling growth and are also defective in the long-day induction of flowering. Consequently, it is postulated that *ATCML24* encodes a potential Ca^2+^ sensor that may enable the response to ABA, dehydration, and ion stresses (Delk *et al.*, 2005). The relationship between calcium signalling, which is facilitated by EF-domain-containing calcium-binding proteins, and root hair development is well established and also involves an additional layer of putative signalling features of the root hair transcriptome, i.e. ROS.

It is well recognized that the ROS signalling network controls a wide range of biological processes, such as growth, development, and responses to biotic and abiotic stimuli. Recent studies have focused on the role that ROS play as signalling molecules rather than on their potential toxicity (Baxter *et al.*, 2014). In order to utilize ROS as signalling molecules, non-toxic levels of ROS in a cell must be maintained when plants are growing under optimal conditions. Any stimuli or stresses can affect the balance between the anticipated ROS production (co-ordinated by ROS-producing enzymes), the unavoidable creation of ROS that occurs during basic cellular processes, and the metabolic counter-process that involves the ROS-scavenging pathways (Mittler *et al.*, 2004; Baxter *et al.*, 2014). In our previous work, we showed that the co-ordinated production of ROS is required for root hair formation in barley (Kwasniewski *et al.*, 2013b). The root-hairless mutant *rhl1.a* is impaired in the peroxidase-dependent production of the hydroxyl radical (•OH) whose accumulation is necessary for the initiation and growth of root hairs. Based on a detailed analysis of ROS production in various root hair mutants in barley, we proposed a model of two-step, co-ordinated ROS formation in root hair cells that involves the root-hair-specific peroxidase(s) and the root-hair-specific NADPH oxidase, which are necessary for proper root hair formation. We also showed that the *rhl1.a* mutant is affected in the expression of two root-hair-specific peroxidase genes, which could presumably coordinate the production of •OH in the WT plants. In the present work, two other peroxidase genes were found to be down-regulated in the roots of the *rhl1.a* mutant, MLOC_15632 and MLOC_49954, which are putative orthologues of the Arabidopsis genes AT5G14130 and AT5G05340, respectively. AT5G14130 encodes a peroxidase that is involved in the response to oxidative stress, and its expression is up-regulated by auxin that is applied externally (indole-3-acetic acid; Goda *et al.*, 2004). AT5G05340 encodes *PEROXIDASE 52* (*AtPrx52*), which contains an ABA response element in its promoter and is up-regulated by ABA treatment but down-regulated in the *Atrpk1* mutant (Osakabe *et al.*, 2005). *RPK1* is involved in the main ABA-mediated signalling pathway and in early ABA perception in Arabidopsis. Interestingly, *AtPrx52* is up-regulated in roots in response to phosphate deprivation (Misson *et al.*, 2005). All of these observations imply that there is an interplay between ABA signalling and ROS production in roots, possibly in response to stress conditions. Analogous complex interconnections between ROS-producing enzymes, ROS level balance, and stress-related signalling have already been shown in studies of the ABA-induced production of ROS in guard cells via the function of RBOHD and RBOHF NADPH oxidases (Zhang *et al.*, 2009).

## Conclusions

- At the onset of water stress, a specific activation of genes regulating processes that are related to water-stress signalling and protection against stress was observed in the roots of WT plants but not in roots of the root-hairless mutant *rhl1.a*.
- On the other hand, at the onset of water stress, the processes of an advanced response to abiotic stimuli, including the response to hydrogen peroxide, heat, and high light intensity, were specifically up-regulated in the leaves of the *rhl1.a* mutant but not in WT plants.
- An extended period of severe stress caused more drastic transcriptome changes in the roots and leaves of the *rhl1.a* mutant than in those of the WT. Likewise, the extended period of water stress caused much stronger damage to PSII in the *rhl1.a* mutant than in its parent cultivar.
- The function of selected root hair development-related genes is potentially related to sensing and signalling of water stress. Taking into account this supposition and the results of transcriptome analysis presented above, we consider that there is a possible role of root hairs as sensors of environmental conditions.

## Supplementary data

Supplementary data are available at *JXB* online.

**Supplementary Table S1.** A list of 66 down-regulated and 20 up-regulated genes in the roots of the *rhl1.a* mutant in comparison with the WT parent cultivar ‘Karat’ (FC≥3; *P*≤0.05; FDR).

**Supplementary Table S2.** Gene Ontology categories (Biological Processes) over-represented in ‘Karat’- or *rhl1.a*-specific subsets of genes, up- or down-regulated during subsequent stages of the experiment in comparison with control conditions in roots or leaves of both genotypes (corrected *P*<0.01) and lists of genes represented by analysed Gene Ontologies.

**Supplementary Fig. S1.** Results of the gene expression analysis in the roots of the root-hairless mutant *rhl1.a* vs the WT cv. ‘Karat’ variety that was performed using gene-specific RT-qPCR and genome-wide Agilent Barley Gene Expression Arrays.

Supplementary Data
