# Supplementary material for: Transcriptome analysis reveals the role of the root hairs as environmental sensors to maintain plant functions under water-deficiency conditions
Source: J Exp Bot. 2015 Nov 19;67(4):1079–94. doi: 10.1093/jxb/erv498 (PMC4753848; doi:10.1093/jxb/erv498)
Supplement: Supplementary Data [file supp_67_4_1079__index.html]

Transcriptome analysis reveals the role of the root hairs as environmental sensors to maintain plant functions under water-deficiency conditions — Transcriptome analysis reveals the role of the root hairs as environmental sensors to maintain plant functions under water-deficiency conditions — Transcriptome analysis reveals the role of the root hairs as environmental sensors to maintain plant functions under water-deficiency conditions — Supplementary Data 

# Transcriptome analysis reveals the role of the root hairs as environmental sensors to maintain plant functions under water-deficiency conditions

## Supplementary Data

Data files

- Supplementary\_Fig.\_S1.pdf - Supplementary Data
- Supplementary\_table\_S1.xlsx - Supplementary Data
- Supplementary\_Table\_S2.xlsx - Supplementary Data
