# Supplementary material for: Transcriptome analysis reveals the role of the root hairs as environmental sensors to maintain plant functions under water-deficiency conditions
Source: J Exp Bot. 2015 Nov 19;67(4):1079–94. doi: 10.1093/jxb/erv498 (PMC4753848; doi:10.1093/jxb/erv498)
Supplement: Supplementary Data [file supp_erv498_Supplementary_Fig._S1.pdf]

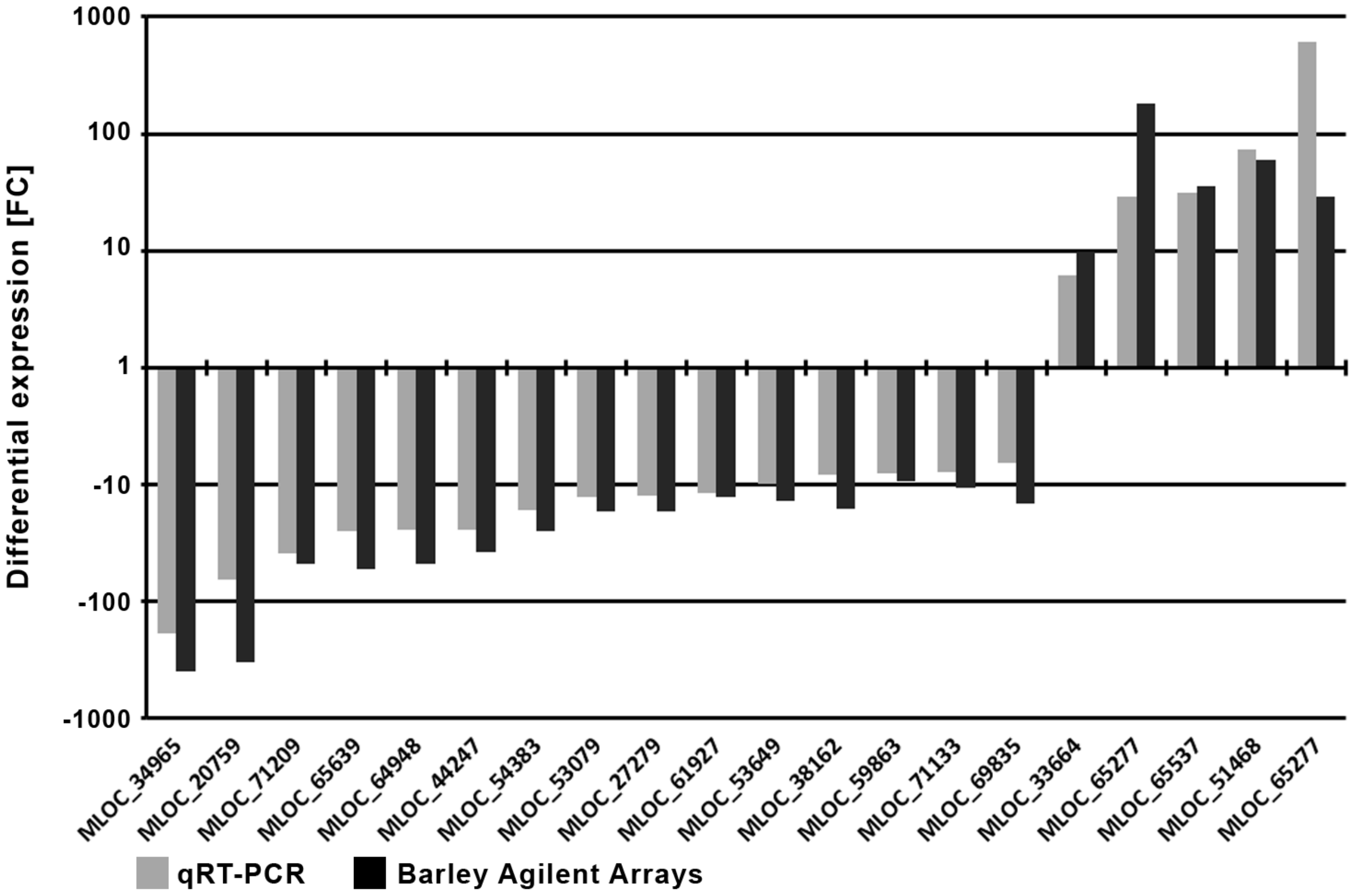

Supplementary Figure S1. Results of the gene expression analysis in the roots of the root hairless mutant *rhl1.a* vs the WT cv. 'Karat' variety that was performed using gene-specific qRT-PCR and genome-wide Agilent Barley Gene Expression Arrays.
